# A Cytosolic Relay of Heat Shock Proteins HSP70-1A and HSP90β Monitors the Folding Trajectory of the Serotonin Transporter*

**DOI:** 10.1074/jbc.M114.595090

**Published:** 2014-09-08

**Authors:** Ali El-Kasaby, Florian Koban, Harald H. Sitte, Michael Freissmuth, Sonja Sucic

**Affiliations:** From the ‡ Institute of Pharmacology, Center of Physiology and Pharmacology, Medical University of Vienna, A-1090 Vienna, Austria and; the § Department of Pharmacology, Faculty of Veterinary Medicine, Mansoura University, 35516 Mansoura, Egypt

**Keywords:** Glycosylation, Heat Shock Protein (HSP), Heat Shock Protein 90 (Hsp90), Serotonin, Serotonin Transporter, Trafficking, Transporter

## Abstract

Mutations in the C terminus of the serotonin transporter (SERT) disrupt folding and export from the endoplasmic reticulum. Here we examined the hypothesis that a cytosolic heat shock protein relay was recruited to the C terminus to assist folding of SERT. This conjecture was verified by the following observations. (i) The proximal portion of the SERT C terminus conforms to a canonical binding site for DnaK/heat shock protein of 70 kDa (HSP70). A peptide covering this segment stimulated ATPase activity of purified HSP70-1A. (ii) A GST fusion protein comprising the C terminus of SERT pulled down HSP70-1A. The interaction between HSP70-1A and SERT was visualized in live cells by Förster resonance energy transfer: it was restricted to endoplasmic reticulum-resident transporters and enhanced by an inhibitor that traps HSP70-1A in its closed state. (iv) Co-immunoprecipitation confirmed complex formation of SERT with HSP70-1A and HSP90β. Consistent with an HSP relay, co-chaperones (*e.g.* HSC70-HSP90-organizing protein) were co-immunoprecipitated with the stalled mutants SERT-R607A/I608A and SERT-P601A/G602A. (v) Depletion of HSP90β by siRNA or its inhibition increased the cell surface expression of wild type SERT and SERT-F604Q. In contrast, SERT-R607A/I608A and SERT-P601A/G602A were only rendered susceptible to inhibition of HSP70 and HSP90 by concomitant pharmacochaperoning with noribogaine. (vi) In JAR cells, inhibition of HSP90 also increased the levels of SERT, indicating that endogenously expressed transporter was also susceptible to control by HSP90β. These findings support the concept that the folding trajectory of SERT is sampled by a cytoplasmic chaperone relay.

## Teaser

**Background:** Naturally occurring mutations in solute carrier 6 (SLC6) family members impair folding of these transporters.

**Results:** Folding-deficient SERT mutants bound heat shock protein (HSP)70-1A, HSP90β, and co-chaperones. Noribogaine synergized with HSP inhibitors in rescuing these SERT mutants.

**Conclusion:** Folding of SERT is assisted by a cytosolic HSP relay.

**Significance:** The folding trajectory of SERT is relevant to folding diseases arising from mutated SLC6 transporters.

## Introduction

Point mutations within the coding sequence of human membrane proteins can result in their retention in the endoplasmic reticulum and thus give rise to clinically relevant phenotypes (1). These are collectively referred to as folding diseases. The mutations aggravate the inherent problems that membrane proteins incur to reach a stable conformation (2). Progress along the folding trajectory is monitored by ER^3^-resident chaperones, which sequentially engage folding intermediates and trigger ER-associated degradation to clear the ER membrane of aberrantly folded proteins and stalled intermediates (3). Transporters of the solute carrier 6 (SLC6) family are of particular interest because mutations give rise to folding diseases that are transmitted in either a recessive or a dominant mode (4). Prominent examples include recessive mutations in the dopamine transporter (SLC6A3) that give rise to a syndrome of infantile dystonia and parkinsonism (5–7), dominant mutations in the norepinephrine transporter (SLC6A2) that cause familial postural hypotension (8), and mutations in glycine transporter 2 (SLC6A5) that result in hyperekplexia/startle disease (9–11). The clinical phenotype of recessive mutations is readily explained by the loss of function: an ER-retained neurotransmitter transporter does not reach the cell surface and in particular presynaptic specialization; hence, released neurotransmitter is not cleared, and vesicular stores are not refilled. The dominant mode of transmission can be rationalized by taking into account that these transporters form oligomers (12). ER export is contingent on oligomerization: transporters that are deficient in oligomerization do not leave the endoplasmic reticulum (13). Accordingly, mutants (8, 14) and transporter fragments (15) retain the wild type transporter in the endoplasmic reticulum and thus act in a dominant negative manner.

It is noteworthy that hyperekplexia/startle disease can result from both recessive (10) and dominant alleles encoding glycine transporter 2/SLC6A5 (9, 11). At the very least, these observations indicate that the folding trajectory of SLC6 transporters is monitored at more than one checkpoint (4). In fact, in the endoplasmic reticulum, SLC6 transporters appear to be shielded by calnexin prior to oligomer formation (16). Similarly, at the other end of the folding trajectory, a mechanism must exist that precludes premature recruitment of coat protein complex II (COPII) components to partially folded transporters. The SEC24 cargo receptor of the COPII machinery binds to an RI/RL motif in the C terminus of SLC6 family members (17–19). Mutations within this motif of SERT do not only impair binding of SEC24; the resulting mutant SERT-R607A/I608A also has a folding defect, which can be remedied by ibogaine (20). Ibogaine binds to the inward facing conformation within the hydrophobic core of SERT (21, 22). Hence, the pharmacochaperoning action of ibogaine, *i.e.* its ability to rescue a folding defect resulting from mutations in the C terminus, suggests that the hydrophobic core and the C terminus cooperate during the folding reaction. This conjecture is also supported by the fact that C-terminal truncations beyond the last 16 amino acids result in inactive SERT variants (20, 23). Thus the proximal segment of the C terminus is required to stabilize the structure of SERT (and of other eukaryotic SLC6 transporters). As long as the C terminus of eukaryotic SLC6 transporters is engaged by cytosolic chaperone proteins, which assist its folding, it is shielded from the COPII machinery. Release of the chaperone proteins signals a stable conformation and renders the C terminus accessible to the cognate SEC24 isoform. Accordingly, in the present study, we searched for chaperone proteins that bound to the C terminus of SERT and of folding-deficient mutants. We identified a stretch of amino acids in the proximal segment of the C terminus that directly interacted with HSP70-1A, visualized the interaction between SERT and HSP70-1A in the endoplasmic reticulum of live cells by Förster resonance energy transfer (FRET), and manipulated the levels and activity of heat shock proteins (HSPs) by siRNA knockdown and with inhibitors, respectively.

## EXPERIMENTAL PROCEDURES

### 

#### 

##### Materials

[^3^H]5-Hydroxytryptamine ([^3^H]5-HT; serotonin; 28.1 Ci/mmol) and [^3^H]imipramine (47.5 Ci/mmol) were purchased from PerkinElmer Life Sciences. Cell culture media, supplements, and antibiotics were from Invitrogen. The Malachite Green Phosphate Assay kit (POMG-25H) was from BioAssay Systems (Hayward, CA). The HSP inhibitors pifithrin-μ (2-phenylethynesulfonamide) and 17-dimethylaminoethylamino-17-demethoxygeldanamycin (17-DMAG) were purchased from Sigma-Aldrich. Noribogaine was donated by Sacrament of Transition (Maribor, Slovenia). Bovine serum albumin (BSA) and Complete^TM^ protease inhibitor mixture were from Roche Applied Science, SDS was from BioMol GmbH (Hamburg, Germany), and Tris and scintillation mixture (Rotiszint®-eco plus) were from Carl Roth GmbH (Karlsruhe, Germany). Anti-GFP antibody (ab290), anti-HSP70-1A antibody (ab47455), anti-HSC70 antibody (ab2788), anti-HSP90α antibody (ab79849), anti-HSP90β antibody (ab53497), anti-HSC70-HSP90-organizing protein (HOP) antibody (ab56873), anti-C terminus of HSP70-interacting protein (CHIP) antibody (ab109103), and anti-p23 antibody (ab2814) were all from Abcam Plc (Cambridge, UK). Protein A-Sepharose and anti-rabbit IgG1 antibody linked to horseradish peroxidase were from Amersham Biosciences. The recombinant purified protein HSP70-1A (ADI-NSP55-D) was purchased from Enzo Life Sciences (Farmindale, NY). All other chemicals were of analytical grade.

##### ATPase Activity Assay

Peptides corresponding to 20 or 24 amino acids of the serotonin transporter C terminus (H_2_N-TPGTFKERIIKSITPETPTE-COOH and H_2_N-RLIITPGTFKERIIKSITPETPTE-COOH, respectively) were produced by David King (University of California, Berkeley). Their identity was confirmed by mass spectrometry. Experiments were performed in assay buffer (30-μl final volume) containing 20 mm Tris-HCl (pH 7.5), 150 mm KCl, 5 mm MgCl_2_, 0.5 mm peptide, 0.5 mm ATP in a 96-well plate. The reaction was started by the addition of 21 pmol of HSP70 or HSC70 and incubated at 37 °C for the indicated time points. The reaction was stopped by adding the malachite green reagent (final volume, 0.1 ml). After 30 min at room temperature, absorbance was measured at 620 nm using a plate reader.

##### Mutagenesis, Cell Culture and Transfections, and Radioligand Binding and Uptake Assays

Mutations were introduced using the QuikChange Lightning site-directed mutagenesis kit (Stratagene Cloning Systems, La Jolla, CA) and the cDNA of human SERT cloned into the pEYFP vector as template. Oligonucleotides were from Operon Biotechnologies (Cologne, Germany). HEK293 cells were grown at 37 °C in a 5% CO_2_ humidified atmosphere in Dulbecco's modified Eagle's medium (DMEM) supplemented with 10% fetal calf serum, 60 mg/liter penicillin, and 100 mg/liter streptomycin. Stably expressing cell lines were grown in complete medium supplemented with geneticin (G418) to maintain selection pressure. JAR cells were propagated in RPMI 1640 medium supplemented with 10% fetal calf serum, 30 mg/liter penicillin, and 50 mg/liter streptomycin. For FRET microscopy, cells were seeded on poly-d-lysine-coated glass coverslips. For siRNAs experiments, HEK293 cells were transfected with 30 nm Silencer Select predesigned siRNAs (s6968 and s6969 for HSP70-1A; s6999 and s7000 for HSP90β; Ambion, Carlsbad, CA) or Silencer Select negative control siRNA (Ambion) using Lipofectamine RNAiMAX reagent according to the protocol provided by the manufacturer. Forty-eight hours following siRNA transfections, the cells were transfected with cDNAs encoding SERT and mutants using Lipofectamine 2000 (Invitrogen). Uptake assays, immunoblotting, and confocal microscopy were done after 24 h. Radioligand binding with [^3^H]imipramine and [^3^H]5-HT uptake assays were carried out as described previously (20).

##### GST Protein Purification and GST Pulldown

The sequence corresponding to the C terminus of SERT was inserted into the pGEX5 vector. Mutations (LII → SSS, LI → SS, R___TPGT, and RLIIT-stop) were introduced as described above. Plasmids were used to transform XL10-Gold *Escherichia coli*. Bacteria (2 liters of liquid culture) were grown at 37 °C to an absorbance at 600 nm (*A*_600_) of 0.6, and then bacteria were transferred to 22 °C and grown to an *A*_600_ of 0.8–0.9. Protein expression was induced at 22 °C by addition of isopropyl 1-thio-β-d-galactopyranoside (0.7 mm) for 3 h. Thereafter, the cells were harvested by centrifugation at 5,000 × *g* for 15 min at 4 °C. Bacterial pellets were resuspended in 25 ml of buffer containing Tris-HCl (pH 8.0), 150 mm NaCl, 0.1% Tween 20 (TBST), and Complete protease inhibitor mixture. Bacteria were incubated with lysozyme (1 mg/ml) for 30 min on ice. Lysates were supplemented with Triton X-100 to a final concentration of 1% and sonicated thrice on ice for 60 s with a Branson Sonifier® cell disruptor B15. The homogenate was cleared from cell debris by centrifugation at 48,000 × *g* for 25 min at 4 °C and incubated for 2 h at 4 °C with 5 ml of glutathione-Sepharose^TM^ 4 Fast Flow (GE Healthcare), which had been pre-equilibrated in TBST, under mild stirring in a glass beaker. The suspension was transferred into chromatography columns, and the Sepharose was washed three times in 50 ml of TBST. Proteins were eluted as 1-ml fractions in 100 mm reduced l-glutathione (Sigma-Aldrich) in TBST (pH 8.0). The protein concentration of each fraction was measured using Bradford reagent. The four fractions with the highest protein concentration were combined and dialyzed against TBST using Spectra/Por® dialysis membranes (Spectrum Laboratories, Inc.). Aliquots were frozen in liquid nitrogen and stored at −80 °C.

For pulldown experiments, GST fusion proteins (10 μg) were immobilized on glutathione-Sepharose (25 μl of packed resin) in 0.15 ml of TBST by gentle rotation for 45 min at room temperature. The Sepharose was recovered by centrifugation and washed by resuspension in 1 ml of TBST. For experiments involving endogenous HSP70, HEK293 cell lysates (0.15 ml) were applied to the Sepharose-bound GST proteins. These cell lysates were prepared as follows. Confluent 10-cm dishes of HEK293 cells were washed twice with ice-cold PBS, the cells were harvested in 1 ml of PBS and centrifuged (5 min at 800 × *g* at 4 °C), the pellets were lysed in 0.5 ml of lysis buffer (20 mm Tris-HCl (pH 7.5), 150 mm NaCl, 5 mm EDTA, 1% Nonidet P-40, Complete protease inhibitor mixture) by gentle rotation at 4 °C for 30 min, and the lysates were centrifuged at 16,000 × *g* for 15 min at 4 °C. Supernatants containing cytosolic components were frozen in liquid nitrogen and stored at −80 °C. For experiments involving recombinant HSP70, 0.15 ml of TBST containing 2 μg of recombinant HSP70 was combined with Sepharose-bound GST proteins. The reactions were carried out by gentle rotation for 60 min at room temperature. The Sepharose beads were recovered by centrifugation and washed thrice with buffer (lysis buffer or TBST, respectively). Bound proteins were released by heat denaturation in 50 μl of sample buffer containing β-mercaptoethanol. Aliquots of the samples (10 μl) were separated by denaturing gel electrophoresis, the resolved proteins were transferred to nitrocellulose membranes, and HSP70 was visualized by immunoblotting.

##### Co-immunoprecipitation

Co-immunoprecipitation experiments were performed as described previously (20) with minor modifications. Detergent lysates were prepared from HEK293 cells stably expressing wild type and mutant versions of SERT in buffer containing 50 mm Tris-HCl (pH 8), 150 mm NaCl, 1% dodecyl maltoside, 1 mm EDTA, protease mixture inhibitors. Alternatively, at the end of the incubation period, HEK293 cells were washed with PBS, and intracellular proteins were cross-linked with 1 mm dithiobis(succinimidyl propionate) in PBS. After 45 min at room temperature, the reaction was quenched by addition of buffer (150 mm NaCl, 100 mm Tris-HCl (pH 7.3)), and the cells were scraped off the dishes, harvested by centrifugation (1,500 × *g* for 5 min at 4 °C), and lysed in 0.1 ml of lysis buffer (150 mm NaCl, 30 mm Tris-HCl (pH 7.3), 5 mm EDTA, 1% Nonidet P-40). The two approaches (*i.e.* immunoprecipitation with or without prior cross-linking) gave equivalent results. The insoluble material was removed by centrifugation at 50,000 × *g* for 30 min at 4 °C. Equal amounts of protein (2 mg/sample) were incubated overnight with 4 μl of anti-GFP antibody (20 μg of IgG). Subsequently, pre-equilibrated protein A-Sepharose (20 mg of protein A/sample) was added and incubated at 4 °C for 5 h with gentle rotation. The beads were collected by centrifugation and then washed three times with lysis buffer. Bound proteins were eluted by denaturation in 0.1 ml of sample buffer containing 40 mm dithiothreitol and 1% mercaptoethanol at 45 °C for 30 min. Aliquots were loaded onto SDS-polyacrylamide gels. After the proteins had been resolved by denaturing electrophoresis, they were transferred to nitrocellulose membranes. Immunoreactive bands were detected by appropriate antibodies (directed against HSPs, co-chaperones, or GFP) and a horseradish peroxidase-conjugated second antibody using enhanced chemiluminescence.

##### FRET Microscopy

FRET was visualized on an epifluorescence Carl Zeiss TM210 microscope using the three-filter method as described previously (24). The plasmid for YFP-HSP70 was generously provided by Harm Kampinga (University of Groningen, Netherlands). Cells were seeded on 29-mm glass bottom dishes and transfected with plasmids encoding YFP-HSP70 (0.2 μg) and wild type or mutant versions of CFP-SERT (0.8 μg). For experiments involving the HSP70 inhibitor VER-155008 (Sigma), the cells were incubated with the drug (40 μm) for 2 h prior to measuring FRET. The images were acquired using a 6× oil immersion objective and an automated filter wheel (Ludl Electronic Products, Hawthorne, NY) to allow for rapid switching between the fluorescence excitation and emission filters for CFP (*I*_CFP_; excitation, 436 nm; emission, 480 nm; dichroic mirror, 455 nm), YFP (*I*_YFP_; excitation, 500 nm; emission, 535 nm; dichroic mirror, 515 nm), and FRET (*I*_FRET_; excitation, 436 nm; emission, 535 nm; dichroic mirror, 455 nm). The images were captured by a charge-coupled device camera and analyzed using the ImageJ PixFRET plug-in (25).

## RESULTS

### 

#### 

##### HSP70 Binds to a DnaK Interaction Motif Located at the C Terminus of SERT

**FIGURE 1.**
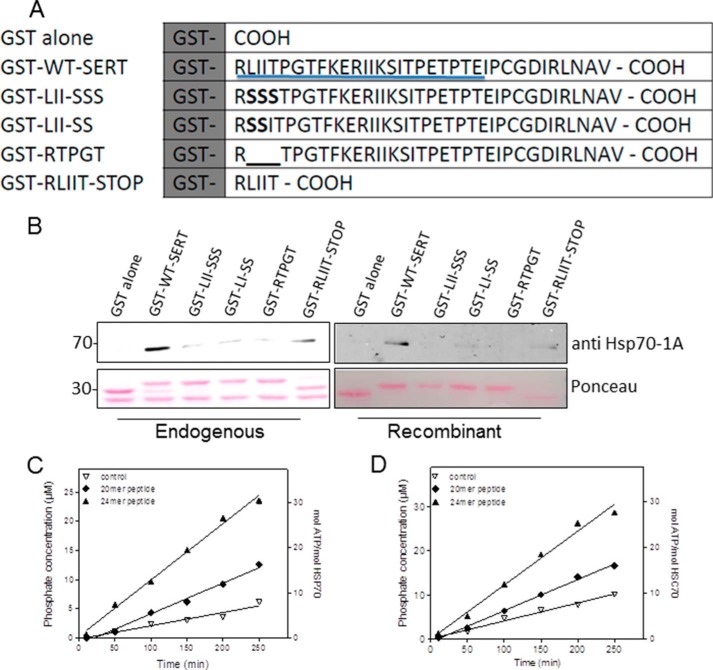
**HSP70 binds to a DnaK interaction motif at the SERT C terminus.** *A*, schematic representation of the GST fusion proteins, which comprised the full length of the C terminus of human SERT starting with Arg^596^ (GST-WT-SERT) and the indicated mutants thereof. *B*, GST pulldown experiments using 10 μg of the indicated GST proteins and either HEK293 cell lysate from ∼2 × 10^6^ cells/sample (*left*) or 2 μg of recombinant HSP70/sample (*right*). Bound proteins were eluted in sample buffer, electrophoretically resolved, and transferred to nitrocellulose membranes to detect immunoreactivity for HSP70-1A. GST fusion proteins were visualized by staining with Ponceau S. *C* and *D*, ATPase assays were carried out with 0.7 μm HSP70 (*C*) or HSC70 (*D*) in the absence (*open triangles*) and presence of 500 μm peptides. The sequence of the 24-mer peptide (*closed triangles*) comprises the residues stretching from Arg^596^ to Glu^620^ in *A* (*underlined* residues); the 20-mer peptide (*closed diamonds*) lacks the first 4 amino acids (RLII). The reaction was started by the addition of ATP (500 μm final concentration) and carried out in a final volume of 30 μl in a 96-well plate for the indicated times at 37 °C. Inorganic phosphate was detected using a malachite green color reaction. Absorbance at 620 nm was measured using a plate reader, and the concentration of inorganic phosphate was measured by comparison with a phosphate standard provided by the manufacturer. Data are from representative experiments that were reproduced ≥3 times.

Inspection of the sequence of SERT reveals the stretch of residues ^595^YRLIITP^601^ adjacent to the last transmembrane helix (TM12) domain that is reminiscent of the canonical sequence NRLLLTG, which is recognized by the bacterial HSP70 homologue DnaK (26, 27). In fact, if the entire amino acid sequence of SERT is searched for putative DnaK binding sites by a computational algorithm (Limbo), the sequence ^595^YRLIITP^601^ is the only candidate binding site within the intracellular portions of SERT. We verified this prediction in pulldown assays with GST fusion proteins comprising the SERT C terminus (35 amino acids) and mutants thereof. These were bound to glutathione-Sepharose and incubated with cell lysates of HEK293 cells (Fig. 1*B*, *left-hand panels*) and purified recombinant HSP70-1A (Fig. 1*B*, *right-hand panels*). Immunoblotting against bound HSP70 showed robust binding between HSP70 and the wild type C terminus of SERT; however, no binding was observed between HSP70 and the GST protein alone (Fig. 1*B*, *cf. first* and *second lanes* in either blot). Mutation of the hydrophobic core region (LII) of the putative binding motif to three serine residues (RSSSTP) or two serines (RSSITP) substantially reduced this interaction (Fig. 1*B*, *cf. third* and *fourth lanes*). We ruled out that introducing serine residues suppressed a nonspecific interaction between HSP70 and the C terminus of SERT by deleting the hydrophobic amino acids LII from the sequence. The resulting GST protein (with the sequence RTPGT at the fusion site) did not bind either cytosolic or purified HSP70-1A (Fig. 1*B*, *fifth lanes*). We also examined whether the mere presence of the ^596^RLIIT^600^ motif sufficed to promote HSP70 binding by creating a GST protein that carried only these five amino acid residues. In fact, GST-RLIIT bound more HSP70-1A than any other mutant (Fig. 1*B*, *right-most lanes*). Taken together, these data indicate that HSP70 interacted directly with the SERT C terminus and that this binding was supported by the RLIIT motif.

HSP70 has a very low intrinsic ATPase activity, which is increased by binding of substrate and of HSP40/J domain proteins (28, 29). Accordingly, we examined whether peptides corresponding to the proximal portion of C terminus were substrates for HSP70 by measuring their effect on ATPase activity on purified recombinant HSP70 (Fig. 1*C*) or recombinant HSC70 (Fig. 1*D*). The 24-mer peptide, which covered residues 596–620 (Fig. 1*A*, *underlined*) stimulated ATPase activity of HSP70 by ∼4-fold (Fig. 1*C*, *triangles*). In contrast, the 20-mer peptide, which lacked the first four residues, was substantially less effective, resulting in ∼2-fold stimulation (Fig. 1*C*, *diamonds*). Similar results were obtained for the constitutively expressed HSP70 isoform HSC70 (Fig. 1*D*).

**FIGURE 2.**
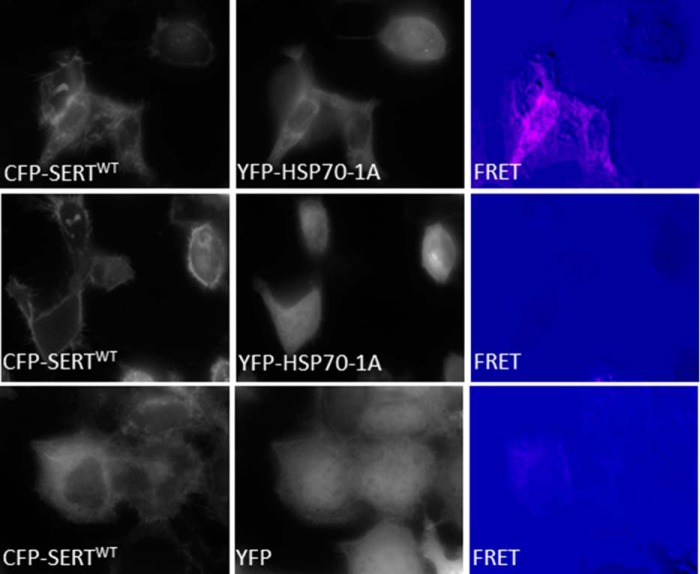
**HSP70 binds SERT exclusively in the ER.** FRET between CFP-SERT YFP-HSP70-1A (*top* and *middle rows*) and YFP (*top row*) is shown. HEK293 cells were seeded onto 29-mm glass bottom dishes and grown to 80% confluence. Cells were transfected with plasmids encoding YFP-HSP70-1A or YFP and CFP-SERT in a ratio of 1:4. FRET was recorded after 24 h using the three-filter method; *i.e.* the *left-hand column* of images was captured at 480 nm after excitation at 436 nm; *in the middle column*, YFP was excited at 500 nm, and the emission was recorded at 500 nm; and in the *right-hand column*, CFP was excited at 436 nm, and the emission was captured at 535 nm. FRET intensity was quantified using the PixFRET software for ImageJ. In the *top* and *middle rows*, cells were selected in which CFP-SERT was predominantly in the ER (*top*) or at the cell surface (*middle*) to illustrate the fact that resonance transfer is only observed if SERT resides in the ER. The experiment is representative of three independent transfections.

Our hypothesis posits that HSP70 assists folding of SERT in the endoplasmic reticulum. Accordingly, we visualized the interaction of YFP-tagged HSP70-1A and CFP-tagged SERT by FRET microscopy in live cells. We specifically compared those transiently transfected HEK293 cells in which copious amounts of CFP-tagged SERT still resided in the endoplasmic reticulum (Fig. 2, *top* and *bottom rows*, *left-hand* images) with cells in which CFP-tagged SERT had already reached the cell surface (Fig. 2, *middle row*). FRET was not detectable if SERT had reached the plasma membrane (Fig. 2, *middle row*). In contrast, FRET was recorded between ER-resident CFP-tagged SERT and YFP-tagged HSP70-1A (Fig. 2, *top row*, *right-hand FRET* image). Because HSP70-1A is a soluble cytosolic protein, we used YFP, which accumulates in the cytosol as a negative control. When expressed to levels comparable with YFP-tagged HSP70-1A, YFP did not give rise to FRET with ER-resident CFP-SERT (Fig. 2, *bottom right panel*).

##### Folding-deficient Mutants of SERT Differ in Their Association with Cytosolic Chaperones HSP70 and HSP90β

**FIGURE 3.**
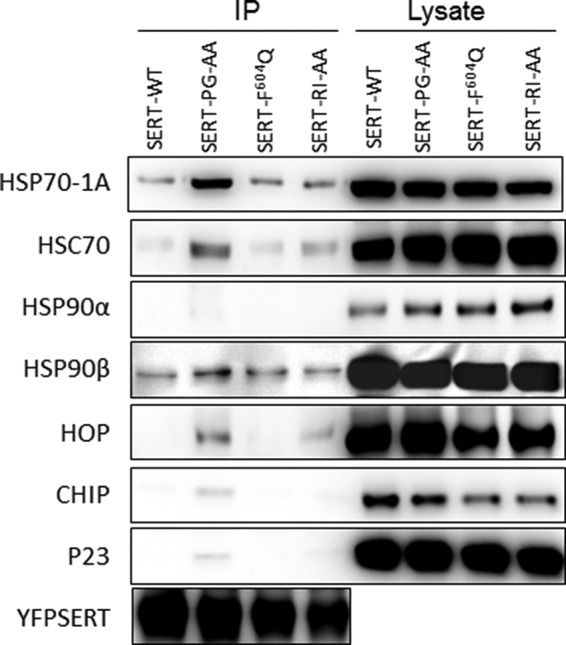
**Association of HSP isoforms and co-chaperones with wild type and folding-deficient mutant SERTs.** Detergent lysates were prepared from HEK293 cells stably expressing YFP-tagged wild type SERT (*SERT-WT*) and the folding-deficient mutants SERT-P601A/G602A (*SERT-PG-AA*), SERT-F604Q, and SERT-R607A/I608A (*SERT-RI-AA*) as described under “Experimental Procedures.” The transporters were immunoprecipitated (*IP*) with an anti-GFP antibody, and immunoreactive bands were detected with appropriate antibodies directed against the indicated HSPs and co-chaperones. The levels of wild type SERT and SERT mutants in the immunoprecipitates were also visualized by blotting for the GFP tag to verify that the difference in associated HSPs and co-chaperones were not accounted for by different levels of transporter protein.

Chaperones of the HSP70 class engage folding intermediates and relay them to an HSP90 isoform (30). We surmised that folding-deficient versions of SERT were stalled at different stages of the folding trajectory and thus ought to associate with HSP. Accordingly, we compared wild type SERT with SERT-P601A/G602A and SERT-R607A/I608A, two folding mutants that were shown previously to differ in the extent to which they were trapped in a calnexin-bound state (20). We also included SERT-F604Q, which we identified during the course of the current work. The folding defect is most severe in SERT-P601A/G602A followed by SERT-R607A/I608A; it is least pronounced in SERT-F604Q (see below). We examined immunoprecipitates of wild type and mutant versions of SERT for the presence of HSP proteins and associated molecules (Fig. 3). HSP70-1A and HSP90β (but not HSP90α) were recovered with SERT. Importantly, the individual mutants of SERT differed in the amount of associated heat shock proteins: immunoprecipitates of SERT-P601A/G602A contained the highest levels of HSP70-1A, HSC70, and HSP90β (Fig. 3, *lane 2*). In addition, these immunoprecipitates also contained the highest levels of HOP, p23/HSP90 co-chaperone, and the E3-ligase CHIP. These observations are consistent with the fact that SERT-P601A/G602A is a stalled folding mutant (20). The co-chaperone HOP assists in the transfer of client proteins between HSP70 and HSP90 isoforms (30). Thus, because the transfer protein HOP was retrieved in the co-immunoprecipitate with SERT-P601A/G602A, the data also support the conjecture that a cytosolic heat shock protein relay assists folding of SERT. In this model, stalled mutants are enriched in HOP because they are relayed back and forth between the HSP70-1A- and the HSP90β-bound state but cannot progress to the native conformation.

**FIGURE 4.**
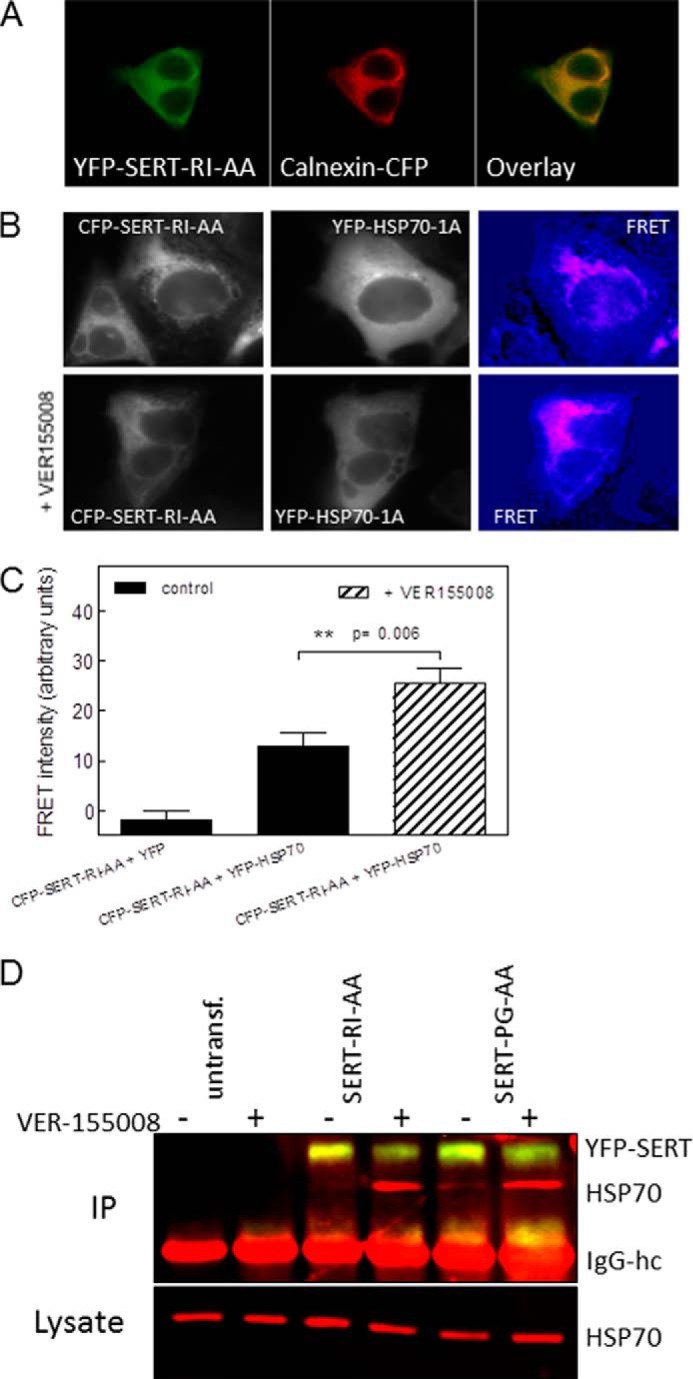
**Trapping of HSP70-1A by the inhibitor VER-155008 enhances the association with ER-resident folding-deficient mutant SERTs.** *A*, CFP-tagged calnexin (*Calnexin-CFP*) was coexpressed with YFP-tagged SERT-R607A/I608A (*YFP-SERT-RI-AA*) in HEK293 cells. CFP and YFP images were captured with a Zeiss LSM 510 confocal microscope (argon laser, 30 milliwatts; helium/neon laser, 1 milliwatt) equipped with an oil immersion objective (Zeiss Plan-Neofluar ×40/1.3) at 458 and 514 nm with 6% laser power and a pinhole size of 2.5 μm. The Zeiss LSM Image Browser (Version 4.2.0.121, Carl Zeiss Microimaging) was used to analyze the images. The merged image (*right panel*) was generated to visualize co-localization of the proteins. *B*, FRET between ER-retained CFP-SERT-R607A/I608A (*CFP-SERT-RI-AA*) and YFP-HSP70-1A in the absence (*top row*) and presence of VER-155008 (*bottom row*). HEK293 cells were transfected, and FRET was recorded as described in the legend to Fig. 2; cells were preincubated with VER-155008 (40 μm) for 2 h prior to capturing the images. *C*, FRET intensities recorded in cells co-transfected with CFP-SERT-R607A/I608A and YFP or YFP-HFSP1A in the absence and presence of VER-155008 were quantified in three independent transfections with 16, 16, and 20 images captured, respectively. The statistical comparison between FRET intensities recorded in the absence and presence of VER-155008 was done using an unpaired Student's *t* test. *Error bars* represent S.E. *D*, untransfected (*untransf.*) HEK293 cells and HEK293 cells expressing YFP-tagged SERT-R607A/I608A (*SERT-RI-AA*) or SERT-P601A/G602A (*SERT-PG-AA*) were incubated in the absence (lanes labeled “−”) and presence of 40 μm VER-155008 (lanes labeled “+”) for 2 h followed by cross-linking. Cellular lysates were prepared and incubated with an antibody directed against GFP to immunoprecipitate SERT. In the *upper* blot, immunoprecipitated (*IP*) SERT and HSP70-1A were simultaneously visualized with antibodies against GFP and HSP70-1A and fluorescent secondary antibodies against murine (*red*) and rabbit IgG (*yellow*). The position of the IgG heavy chain (*IgG-hc*) is also indicated. The cellular levels of HSP70-1A were determined by blotting an aliquot (0.5%) of the lysate (*lower* blot). The experiment was reproduced twice with similar results.

The folding deficiency of SERT-R607A/I608A is less pronounced than that of SERT-P601A/G602A. However, appreciable levels of HSP70-1A and of HOP were still co-immunoprecipitated with SERT-R607A/I608A (Fig. 3, *fourth set* of *lanes*). Accordingly, we reasoned that the association of SERT-R607A/I608A with HSP70-1A may be enhanced by trapping the complex. We first verified that SERT-R607A/I608A was confined to the endoplasmic reticulum by examining its co-localization with calnexin. It is evident from the confocal imaging in Fig. 4*A* that YFP-tagged SERT-R607A/I608A and CFP-tagged calnexin were strictly co-localized. We then made use of VER-155008. This adenosine analog competitively inhibits HSP70 with an IC_50_ value of 500 nm, disrupts the cycle of HSP70 association and dissociation, and blocks it in a state that is halfway between the closed (ADP-liganded) and the open state (31). VER-155008 is therefore predicted to promote the steady-state association between HSP70-1A and the ER-trapped mutant SERT-R607A/I608A. This prediction was verified by recording FRET between CFP-tagged SERT-R607A/I608A and YFP-tagged HSP70 in live cells (Fig. 4*B*). A statistical comparison showed that there was a statistically significant increase in resonance energy transfer in cells that had been treated with VER-155008 (Fig. 4*C*). We ruled out that the increased association of SERT-R607A/I608A and YFP-tagged HSP70-1A was due to a change in the expression levels of endogenous HSP70-1A: if HEK293 cells were pretreated with VER-155008 for 2 h (*i.e.* the conditions used for FRET), then the compound also promoted the association of endogenous HSP70-1A with SERT-R607A/I608A and SERT-P601A/G602A (Fig. 4*D*, *cf. lanes* labeled “+” and “−” in the *upper* blot). However, the levels of endogenous HSP70-1A were comparable in control cells and VER-155008-treated cells regardless of whether the HEK293 cells expressed the mutants SERT-R607A/I608A and SERT-P601A/G602A or not (Fig. 4*D*, *lower* blot).

##### Depletion or Inhibition of HSP70 and HSP90 Differentially Affects Misfolded SERT Mutants

**FIGURE 5.**
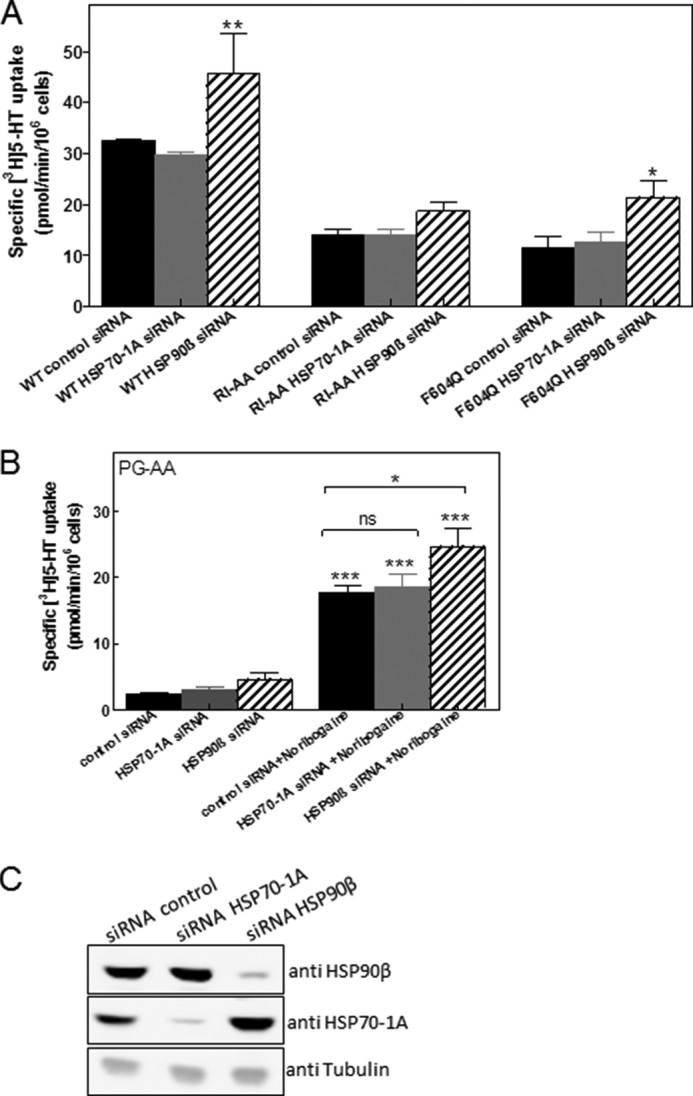
**Effect of siRNA-induced depletion of HSP70-1A and of HSP90β on the surface level of SERT and folding-deficient SERT mutants.** HEK293 cells were transfected with 30 nm siRNAs to deplete HSP70-1A or HSP90β or with scrambled siRNA (negative control). After 48 h, the cells were transfected with the plasmids encoding wild type SERT (*WT*), SERT-F604Q (*F604Q*), SERT-R607A/I608A (*RI-AA*), and SERT-P601A/G602A (*PG-AA*). Twenty-four hours after this second transfection, surface levels of the transporters were quantified by measuring specific [^3^H]5-HT uptake (*A* and *B*). *Error bars* represent S.E. Aliquots of the cells were lysed, and the levels of HSP70-1A and HSP90β in the lysates (20 μg/lane) were determined by immunoblotting (*C*). Cells expressing SERT-P601A/G602A were also preincubated with noribogaine (10 μm) to pharmacochaperone the mutated transporter (*right-hand* set of *bars* in *B*). Data are from at least three independent experiments carried out in triplicate. Statistically significant differences were evaluated by one-way analysis of variance followed by Tukey's post hoc *t* tests (in *A*, *, *p* < 0.05; **, *p* < 0.01 *versus* scrambled control siRNA; in *B*, ***, *p* < 0.001 *versus* the corresponding uptake in the absence of noribogaine; *ns*, not significant; *, *p* < 0.05 for the indicated comparisons).

The recently proposed chaperone-COPII exchange model posits that the SEC24 binding site becomes accessible upon release of HSP90 (4). Accordingly, manipulations in HSP levels may affect the steady-state levels of SERT at the cell surface. The levels of HSP70-1A and HSP90β were lowered by introducing siRNA into HEK293 cells (Fig. 5*C*), and SERT and mutants thereof were subsequently transiently expressed in these cells. Specific [^3^H]5-HT uptake was used to quantify cell surface levels of SERT because only transporters located at the plasma membrane can take up the substrate: depletion of HSP90β, but not of HSP70-1A, resulted in a statistically significant increase in substrate uptake by SERT (Fig. 5*A*, *left-hand* set of *bars*). This is consistent with the chaperone-COPII exchange model: the HSP relay terminates in HSP90, the release of which is (at least in part) rate-limiting for recruitment of SEC24. Likewise, depletion of HSP90β also enhanced the ability of SERT-F604Q to transport [^3^H]5-HT (Fig. 5*A*). In contrast, neither transport by SERT-R607A/I608A (Fig. 5*A*) nor by SERT-P601A/G602A was affected by depletion of HSP90β in a statistically significant manner (Fig. 5*C*). This may reflect quantitative rather than qualitative differences because depletion of HSP90β also resulted in a numerical increase in transport by these two mutants.

**FIGURE 6.**
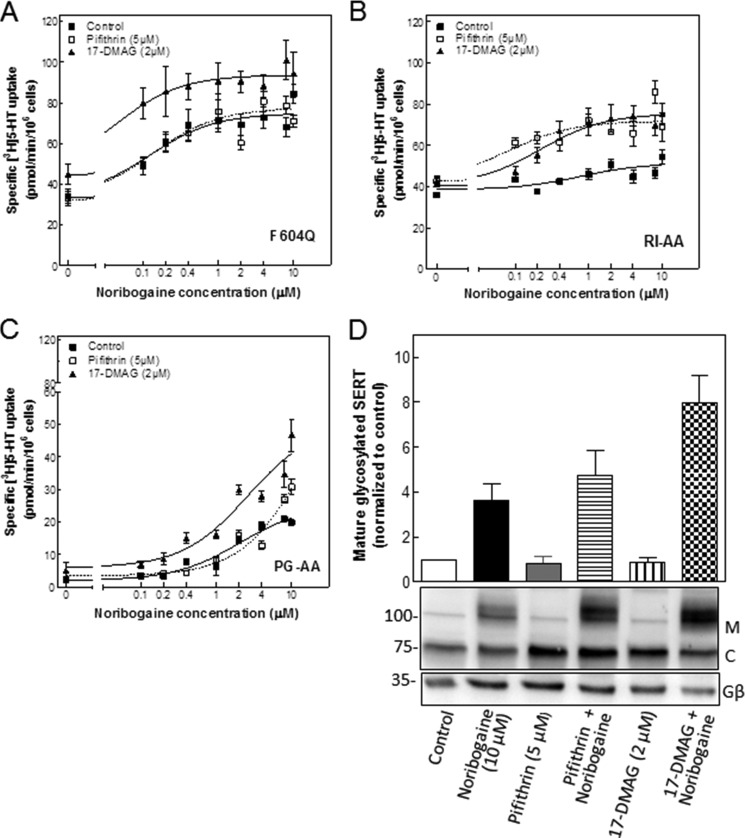
**HSP inhibitors synergize with the pharmacochaperone ibogaine in rescuing the folding-deficient SERT mutants SERT-F604Q (*A*) and SERT-R607A/I608A (*B*) but not SERT-P601A/G602A (*C*).** *A–C*, HEK293 cells stably expressing YFP-tagged SERT-F604Q (*F604Q*; *A*), SERT-R607A/I608A (*RI-AA*; *B*), and SERT-P601A/G602A (*PG-AA*; *C*) seeded onto 48-well plates and incubated in the absence (*Control*; *closed square*s) and presence of 17-DMAG (2 μm; *closed triangles*) or pifithrin-μ (5 μm; *open squares*) with the indicated concentrations of noribogaine for 24 h. Cell surface expression of the transporters was quantified by measuring specific [^3^H]5-HT uptake. Data are from three to four independent experiments done in triplicate; *error bars* indicate S.E. The curves were generated by fitting the data points to the equation of a rectangular hyperbola. *D*, detergent lysates were prepared from HEK293 cells expressing YFP-tagged SERT-P601A/G602A, which had been incubated in the absence (*Control lane*) and presence of noribogaine (10 μm), pifithrin-μ (5 μm), a combination of noribogaine and pifithrin-μ, 17-DMAG (2 μm), and a combination of noribogaine and 17-DMAG for 24 h. After electrophoretic separation and transfer to nitrocellulose membranes, immunoreactivity of SERT was detected via its N-terminal tag with an antibody directed against GFP (*upper* blot). *C* and *M* indicate the position of the core glycosylated (ER-resident) and mature (Golgi and post-Golgi) forms of SERT, respectively. The blot was also probed with an antibody recognizing Gβ subunits as a loading control (*lower* blot). The bar diagram represents the densitometric quantification of the mature fully glycosylated (*M*) corrected for loading. The intensity seen under control conditions was set to 1, and the -fold change observed in the presence of compounds was averaged from three independent experiments (means ± S.E.).

ER-trapped mutants can be rescued by various chemical and pharmacological chaperones (20). In our experiments, we opted for the ibogaine metabolite noribogaine (12-hydroxyibogamine), which has an ∼10-fold higher affinity for SERT and is more effective as pharmacochaperone than ibogaine (data not shown). Noribogaine can also rescue SERT-P601A/G602A (*cf*. Figs. 5*B* and 6*C*). We combined siRNA-mediated depletion of HSP90β with noribogaine based on the assumption that pharmacochaperoning by noribogaine resulted in advancing SERT-P601A/G602A along its folding trajectory. Thus, noribogaine was predicted to render SERT-P601A/G602A susceptible to knockdown of HSP90β. This was the case (Fig. 5*B*, *right-hand* set of *bars*).

As a complementary approach, we also explored the pharmacochaperoning action of noribogaine in the absence and presence of HSP inhibitors: HEK293 cells stably expressing the mutated versions of SERT were treated with increasing concentrations of the pharmacochaperone noribogaine (0.1–10 μm) in the presence or absence of the HSP70 inhibitor pifithrin-μ (32) or of the HSP90 inhibitor 17-DMAG (33). We separately verified by using radiolabeled imipramine binding that pifithrin-μ and 17-DMAG did not bind to SERT directly (data not shown). Pretreating cells with noribogaine for 24 h resulted in a concentration-dependent increase in surface levels of mutated SERT as quantified by determining specific [^3^H]5-HT uptake (Fig. 5): the EC_50_ values of noribogaine were lowest for SERT-F604Q followed by SERT-R607A/I608A and SERT-P601A/G602A (Table 1). Importantly, the individual mutants differed substantially in their susceptibility to the combined action of noribogaine and HSP inhibitors: in SERT-F604Q-expressing cells, the concentration-response curve was shifted to the left upon inhibition of HSP90β by 17-DMAG but not upon inhibition of HSP70-1A by pifithrin-μ (Fig. 6*A* and Table 1). In contrast, both 17-DMAG and pifithrin-μ sensitized SERT-R607A/I608A to the pharmacochaperoning action of noribogaine (Fig. 6*B* and Table 1). The difference in synergism indicated that SERT-R607A/I608A and SERT-F604Q were stalled in states in which progress was limited by HSP70 and HSP90 and solely by HSP90, respectively. In SERT-P601A/G602A, inhibition of HSP90 by 17-DMAG augmented the level of active transporter at the cell surface uniformly at each concentration of noribogaine, resulting in an upwardly shifted concentration-response curve (Fig. 6*C*) with a comparable EC_50_ (Table 1). The lack of synergism between noribogaine and 17-DMAG suggested an additional rate-limiting step prior to release of HSP90β. This conjecture was confirmed by examining the effect resulting from HSP70 inhibition by pifithrin-μ, which only enhanced the action of high concentrations of noribogaine (Fig. 6*C*, *open squares*). Accordingly, in the presence of pifithrin-μ, higher concentrations of noribogaine were required to elicit a half-maximum effect (Table 1). Thus, by contrast, with SERT-R607A/I608A and SERT-F604Q, SERT-P601A/G602A appeared to be stalled at an early stage of the folding trajectory. Accordingly, inhibition of HSP70 precluded progress through the folding trajectory, and an excess of noribogaine was required to functionally rescue the mutant. We confirmed that noribogaine and the combination of HSP inhibitors and noribogaine affected maturation of SERT-P601A/G602A by examining the proportion of immunoreactivity migrating at about 75 and 90 kDa (Fig. 6*D*): the lower (labeled *C*) and the upper bands (labeled *M*) were shown previously to correspond to the ER-resident, core glycosylated transporter and the mature glycosylated transporter, respectively (20). In the absence of any inhibitor, essentially all immunoreactivity of SERT-P601A/G602A was confined to the ER-resident, core glycosylated band (Fig. 6*D*, *first lane*). Noribogaine increased the mature glycosylated form (*M*) (Fig. 6*D*, *second lane*); the HSP inhibitors *per se* did not have any appreciable effect but augmented the action of 10 μm noribogaine with 17-DMAG being more effective than pifithrin-μ (Fig. 6*D*, *third* and *fifth lanes*, respectively). We also blotted for the levels of G protein β-subunits (Fig. 5*D*, *lower* blot) using an antiserum that recognizes all Gβ isoforms (34). This verified that the observed increases in mature SERT-P601A/G602A were not accounted for by unequal loading. Thus, the combinations of noribogaine and pifithrin-μ or 17-DMAG recapitulated the effect seen when quantifying surface expression by substrate uptake (Fig. 6*C*, *cf*. data points at 10 μm noribogaine). Analogous observations were made with SERT-F604Q and SERT-R607A/I608A (data not shown).

**TABLE 1:** **Shift in the EC_50_ of the pharmacochaperone noribogaine induced by inhibition of HSP70 or of HSP90** HEK293 cells stably expressing ER-retained SERT mutants SERT-P601A/G602A, SERT-F604Q and SERT-R607A/I608A were treated with increasing concentrations of noribogaine (0.1–10 μm) in the absence or presence of pifithrin-μ (5 μm) or of 17-DMAG (2 μm) as outlined in Fig. 5. After 24 h, specific [^3^H]5-HT uptake was measured as described under “Experimental Procedures.” The EC_50_ values are shown as geometric means with 95% confidence limits from three individual experiments, each performed in triplicate. The statistical comparison was done by analysis of variance followed by Tukey's post hoc test.

SERT mutant	EC_50_ of noribogaine for enhancing substrate uptake		
	Noribogaine alone (control)	Noribogaine + 17-DMAG (2 μm)	Noribogaine + pifithrin (5 μm)
	μ*m*		
SERT-P601A/G602A	2.06 (1.08–3.93)	2.63 (1.22–5.70)	12.1*^a^* (4.7–31.5)
SERT-R607A/I608A	0.78 (0.12–5.22)	0.22*^b^* (0.08–0.63)	0.08*^c^* (0.02–0.28)
SERT-F604Q	0.12 (0.05–0.31)	0.04*^b^* (0.01–0.20)	0.13 (0.06–0.29)

*^a^ p* < 0.001, significantly different from control.

*^b^ p* < 0.05, significantly different from control.

*^c^ p* < 0.01, significantly different from control.

##### Complex Formation between SERT and HSP Is Altered by Pharmacochaperoning

**FIGURE 7.**
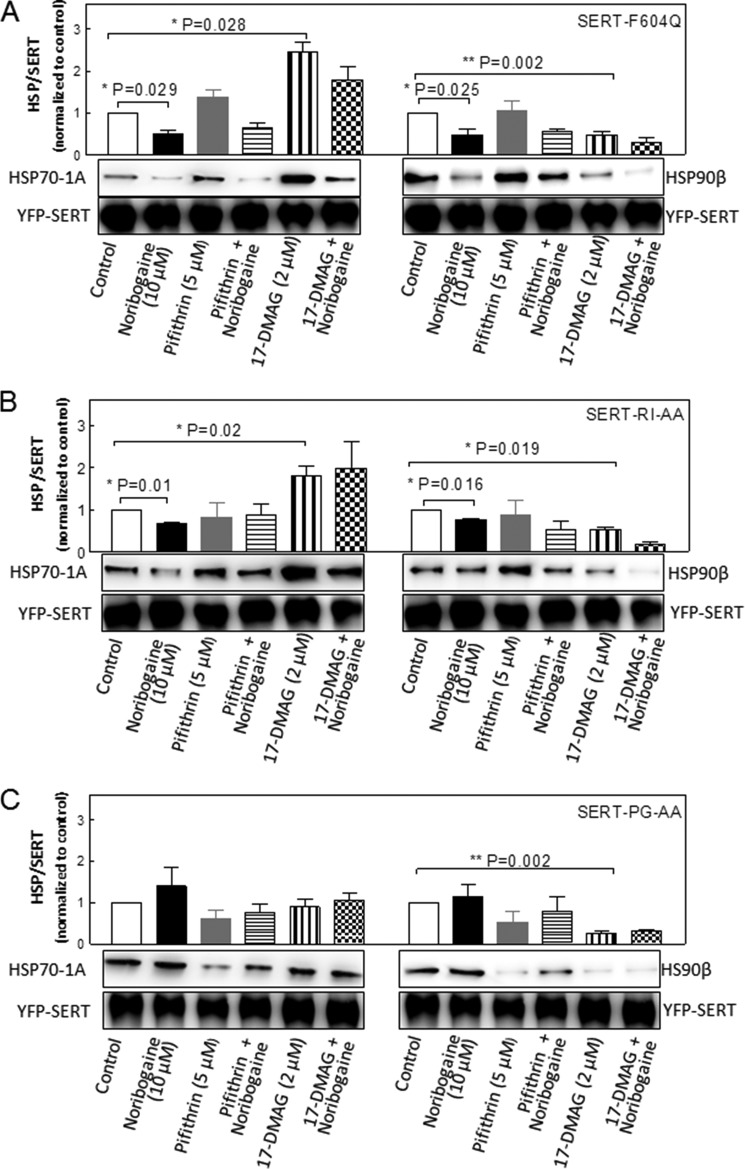
**Co-immunoprecipitation of HSP70-1A and of HSP90β with SERT-F604Q (*A*), SERT-R607A/I608A (*B*), and SERT-P601A/G602A (*C*).** HEK293 cells stably expressing YFP-tagged SERT-F604Q (*F604Q*; *A*), SERT-R607A/I608A (*SERT-RI-AA*; *B*), and SERT-P601A/G602A (*SERT-PG-AA*; *C*) were incubated in the absence (*Control lane*) and presence of noribogaine (10 μm), pifithrin-μ (5 μm), a combination of noribogaine and pifithrin-μ, 17-DMAG (2 μm), and a combination of noribogaine and 17-DMAG for 24 h. Detergent lysates were prepared from these cells, and SERT mutants were immunoprecipitated with an antibody directed against GFP as outlined under “Experimental Procedures.” Immunoreactive bands were detected by appropriate antibodies directed against HSP70-1A, HSP90β, or GFP. Integrated density was quantified using ImageJ 1.43 and used to calculate the ratio of HSP over SERT (GFP) immunoreactivity. The ratios were then normalized to control. Bar diagrams represent the means ± S.E. (*error bars*) from three to four experiments. The data were analyzed for statistically significant differences by analysis of variance or Friedman test followed by the appropriate post hoc test. For the sake of clarity, only those statistically significant differences relevant to the discussion are highlighted (see pertinent section in text).

In the HSP relay, folding intermediates advance from an HSP70-bound state to conformations engaging HSP90 (30). Pharmacochaperoning of SERT mutants by noribogaine is predicted to shift the equilibrium in favor of the stable mature conformation and thus to affect complex formation with HSP70-1A and HSP90β provided that the mutants are stalled within the HSP relay. Accordingly, we immunoprecipitated SERT-F604Q (Fig. 7*A*), SERT-R607A/I608A (Fig. 7*B*), and SERT-P601A/G602A (Fig. 7*C*) from cells that had been treated with noribogaine and HSP inhibitors and examined the association with HSP70-1A and HSP90β. Inhibition of HSP90 by 17-DMAG uniformly decreased the association of SERT mutants with HSP90β (Fig. 7, *A–C*) but increased the association with HSP70-1A. This was to be expected if these mutants were stalled within the relay. By contrast with SERT-F604Q and SERT-R607A/I608A, blockage of HSP90 by 17-DMAG did not promote the association SERT-P601A/G602A with HSP70-1A (Fig. 7*C*). Similarly, inhibition by pifithrin-μ caused a decline in SERT-P601A/G602A, but not of SERT-F604Q and of SERT-R607A/I608A, complexed to HSP90β. This is consistent with the conclusion that SERT-P601A/G602A is trapped at an early stage, *i.e.* prior to the HSP70-HSP90 relay. Accordingly and contrary to SERT-F604Q (Fig. 7*A*) and SERT-R607A/I608A (Fig. 7*B*), noribogaine did not reduce complex formation of SERT-P601A/G602A with HSP70-1A and HSP90β (Fig. 7*C*) presumably because it increased the flux of folding intermediates through the folding trajectory and thus promoted the association of SERT-P601A/G602A with HSPs.

##### HSP90β Also Controls Levels of Endogenously Expressed SERT

**FIGURE 8.**
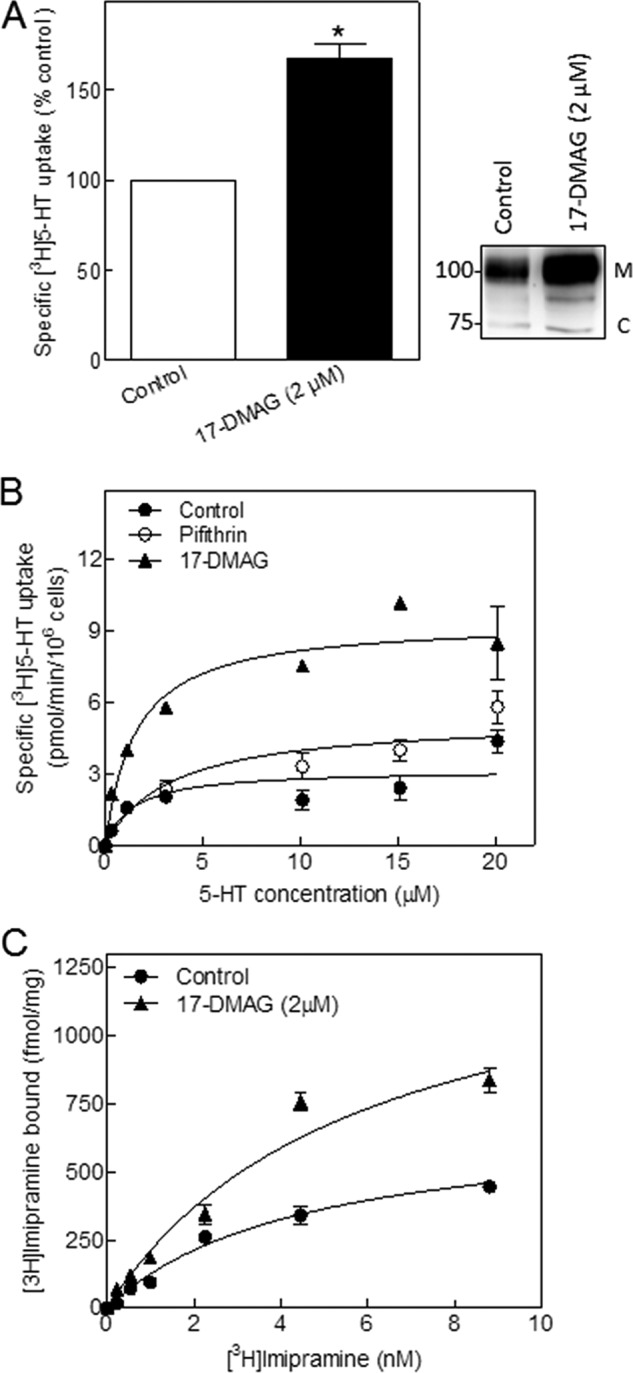
**Inhibition of HSP90 leads to enhanced surface expression of SERT protein.** *A*, HEK 293 cells stably expressing YFP-tagged wild type SERT were seeded in 48-well plates and incubated in the absence (*Control*) and presence of 17-DMAG (2 μm) for 24 h. Surface levels of SERT were quantified by measuring specific [^3^H]5-HT uptake (*right-hand panel*); uptake by untreated control cells was set to 100% and corresponded to 59 ± 2.4 pmol/10^6^ cells/min (*n* = 7). The statistical significance of the difference was assessed by a paired Wilcoxon test (*p* = 0.016). Detergent lysates were prepared from these cells, electrophoretically resolved, and transferred to nitrocellulose membranes, and immunoreactive SERT was detected via its N-terminal tag with an antibody directed against GFP (*right-hand panel*). *C* and *M* indicate the position of the core glycosylated (ER-resident) and mature (Golgi and post-Golgi) forms of SERT, respectively. *B*, JAR cells, which endogenously express SERT, were seeded in 48-well plates and incubated in the absence (*Control*; *closed circles*) and presence of 17-DMAG (2 μm; *closed triangles*) or pifithrin-μ (5 μm; *open circles*). After 24 h, the indicated concentrations of [^3^H]5-HT were added, and specific uptake was determined over a 5-min incubation period. *C*, membranes (40 μg/assay) were prepared from JAR cells that had been maintained in the absence (*Control*; *closed circles*) and presence of 17-DMAG (2 μm; *closed triangles*) for 24 h and incubated in the presence of the indicated concentrations of [^3^H]imipramine for 30 min at 30 °C. Nonspecific binding was determined in the presence of 10 μm paroxetine and was subtracted. Data in *B* and *C* are means ± S.E. (*error bars*) from three independent experiments done in triplicate (*B*) or duplicate (*C*). The curves were generated by fitting the data points to a rectangular hyperbola.

Heterologously expressed wild type SERT was also retrieved in complex with HSP90β (see Fig. 3*A*), and siRNA-mediated depletion of HSP90β resulted in increased cell surface levels as assessed by substrate uptake (see Fig. 5*A*). Accordingly, we also verified that inhibition of HSP90 increased the surface levels of the transporter. In HEK293 cells, which stably expressed wild type SERT, pretreatment with 17-DMAG enhanced substrate uptake (Fig. 7*A*, *left-hand panel*) and raised the level of the mature form of the transporter (Fig. 8*A*, *right-hand panel*, *M* band). It was of obvious interest to recapitulate these findings with endogenously expressed SERT. Hence, we incubated the choriocarcinoma cell line JAR with 17-DMAG. These cells endogenously express SERT. Pretreatment of JAR cells with 17-DMAG substantially increased both maximal uptake velocity (*V*_max_) of 5-HT (Fig. 8*B*) and binding of the SERT-specific radioligand [^3^H]imipramine (Fig. 8*C*) without affecting the affinity for substrate or radioligand (Table 2). Thus, blockage of HSP90 by 17-DMAG increased the steady-state levels of SERT in JAR cells. In contrast, inhibition of HSP70 by pifithrin-μ did not have any detectable effect (Fig. 8*B* and Table 2).

**TABLE 2:** **Effects of HSP70 and HSP90 inhibitors on serotonin uptake by imipramine saturation binding to JAR cells** JAR cells were incubated in the absence (control) or presence of pifithrin-μ (5 μm) or of 17-DMAG (2 μm). After 24 h, specific [^3^H]5-HT uptake and [^3^H]imipramine binding were measured as outlined in Fig. 7. The *K_m_* and *V*_max_ values are shown as arithmetic means ±S.E. from three independent experiments performed in triplicate. Analysis of variance followed by Tukey's post hoc test was used to evaluate differences between individual conditions for 5-HT uptake. For the imipramine binding experiment, the comparison was done by paired *t* test. ND, not determined.

Parameter	Treatment		
	Control	Pifithrin (5 μm)	17-DMAG (2 μm)
5-HT *K_m_* for 5-HT uptake (μm)	2.01 ± 0.37	2.23 ± 0.04	2.00 ± 0.26
*V*_max_ of 5-HT uptake			
(pmol/10^6^ cells/min)	3.21 ± 0.54	4.64 ± 0.44	10.64 ± 0.81*^a^*
*K_D_* for binding of [^3^H]imipramine (nm)	4.84 ± 0.18	ND	5.61 ± 0.18
*B*_max_ for binding of [^3^H]imipramine (fmol/mg)	691.10 ± 1.96	ND	1480 ± 69.34*^b^*

*^a^ p* < 0.05, significantly different from control.

*^b^ p* = 0.005, significantly different from control.

## DISCUSSION

When compared with their bacterial orthologs, eukaryotic SLC6 transporters have extended N and C termini. These extensions reflect the complexity of their regulation because they allow for the recruitment of interacting proteins and thus for modulatory input. This is presumably more important in a compartmentalized cell (in particular if it resides in a multicellular organism) than in bacterial cells. In fact, many regulatory proteins are known to bind to the N or C terminus of mammalian SERT (for a review, see Ref. 35). Prominent examples of signaling proteins that bind to the C terminus include calcineurin (36) and neuronal NO synthase (37). In addition, the C terminus of SERT plays an important role in folding (20) and ER export because it harbors the binding site for the cargo receptor SEC24C (18, 19). Based on these observations and related findings with the G protein-coupled A_2A_-receptor (38), a chaperone-COPII exchange model was proposed (4). This model posits that cytosolic chaperones engage the C terminus of ER-resident folding intermediates of the transporter; if the transporter reaches a stable conformation, the chaperones are released, and only then can the cargo receptor of the COPII coat (*i.e.* SEC24C) be recruited to the C terminus of the receptor. Conversely, if the transporter is stalled in a misfolded state, the chaperones reroute it to ER-associated degradation. Our experiments were designed to explore the involvement of cytosolic chaperones in the folding trajectory of SERT. They demonstrate that HSP70-1A and HSP90β associate with SERT, assist in its folding in a relay, and thus determine its availability for ER export. This conclusion is based on the following findings. (i) SERT has a canonical DnaK/HSP70 binding motif in the proximal portion of its C terminus. (ii) In live cells, the interaction between HSP70-1A and SERT was confined to ER-resident rather than to surface-expressed transporters. (iii) Complex formation of SERT and in particular folding-deficient mutants of SERT with HSP70-1A was confirmed by co-immunoprecipitation. (iv) The association with HSP70-1A was *per se* not limiting for ER export of SERT: SERT and SERT mutants were relayed to HSP90β. Accordingly, co-chaperones (HOP, p23, and CHIP) were recovered in the immunoprecipitates of stalled SERT mutants. Depletion of HSP90β by siRNA or its inhibition by 17-DMAG increased the cell surface expression of wild type SERT but was devoid of any action on the more severely affected folding-deficient mutants SERT-R607A/I608A and SERT-P601A/G602A. (v) These mutants were rendered susceptible to the inhibition of HSP70 and HSP90 by pharmacochaperoning with noribogaine: this not only increased surface levels and the mature form of the transporter, but it also altered the level of HSP70-1A and/or HSP90β in a manner consistent with a shift in steady-state distribution of folding intermediates. (vi) Finally, in JAR cells, inhibition of HSP90 also increased the levels of the transporter SERT, indicating that endogenously expressed SERT was also susceptible to control by HSP90β. Taken together, these findings support the concept that the folded state of SERT is sampled by a cytoplasmic chaperone relay prior to recruitment of the COPII coat to the C terminus of SERT.

Folding of transmembrane proteins is a challenge because it occurs in three compartments: *i.e.* the aqueous phase of the ER lumen, the lipid phase of the membrane, and the aqueous milieu of the cytosol. Because of its medical relevance, the most extensively studied folding problem is that incurred by folding-deficient mutants of cystic fibrosis transmembrane conductance regulator (CFTR; ABCC7), specifically its most prevalent representative CFTR-ΔF508. The folding state of CFTR-ΔF508 is monitored by a cytoplasmic heat shock protein relay (39); surface expression of CFTR-ΔF508 can be restored by targeting this relay (40). Arguably, the nucleotide binding domains of ABC transporters represent large intracellular domains that must adopt a complex fold. Hence, it is not surprising that cytoplasmic chaperones assist in the folding trajectory of these proteins. However, there are examples where other membrane proteins (in particular members of the SLC family of transporters) were identified as clients of cytosolic heat shock proteins: folding of the renal NaCl transporter (SLC12A3) is for instance assisted by HSP70 and HSP90, and clinically relevant mutants that lead to Gitelman syndrome are triaged by HSP70-1A (41). It is worth noting that inhibition of HSP90 does not necessarily augment cell surface levels: in fact, cell surface expression of SLC12A3 is reduced by treating cells with geldanamycin analogs (41), whereas that of pendrin (SLC26A4) is enhanced (42). The reason for this discrepancy is not clear. However, it is unrelated to the nature of the HSP90 isoform involved. The A_2A_-receptor interacts specifically with HSP90α but not with HSP90β (43); in contrast, SERT was an exclusive client of HSP90β. Yet in both SERT and the A_2A_-receptor blockage or depletion of the pertinent HSP90 isoform resulted in increased surface levels of the client protein. Possibly the outcome of HSP90 inhibition depends on the steady-state distribution of folding intermediates along the folding trajectory. In this hypothetical model, surface expression of clients that can reach the folded state in the absence of HSP90 is augmented upon HSP90 inhibition or depletion. Circumstantial evidence to support this conjecture is provided by the synergism that was observed between noribogaine and HSP90 inhibition for SERT-F604Q and SERT-R607A/I608A and the absence thereof in SERT-P601A/G602A. From a teleological perspective, it is consistent with stringent ER quality control that proteins that can achieve stable conformation in the absence of assistance by HSP90 are nevertheless subjected to conformational sampling by HSP90 prior to recruitment of the COPII coat. This provides an additional safeguard against ER export of partially folded transmembrane proteins, which would be prone to aggregation and thus wreak havoc at the cell surface.

Calcineurin/protein phosphatase 2B targets the same binding site that we identified for HSP70-1A (36); therefore it is not surprising that the same or overlapping docking site(s) are engaged by several proteins. Although we cannot formally rule out that calcineurin may also play a role in assisting ER export of SERT, this appears unlikely for several reasons. Calcineurin was co-immunoprecipitated with the mature form of SERT, and the association of calcineurin with SERT resulted in dephosphorylation of its N terminus and counteracted internalization of SERT driven by protein kinase C. It is therefore more plausible that calcineurin enhances SERT surface levels by modulating regulated and constitutive internalization of SERT (44) rather than by enhancing ER export. Conversely, the interaction of HSP70-1A with SERT was confined to the ER-resident form and not seen with surface-expressed transporter. This can be rationalized by assuming that the binding site is not accessible to HSP70-1A if SERT has adopted its stable conformation.

Our observations are of relevance for diseases that arise from misfolded SLC6 transporters (4). The most severe clinical manifestations result from mutations in SLC6A3, SLC6A5, SLCA18, and SLCA19 giving rise to infantile dystonia and parkinsonism (5–7), hyperekplexia (9–11), and Hartnup disease (45–47), respectively. Understanding the folding trajectory of individual transporters and the associated chaperone relay may open avenues to restoring the function of ER-retained mutants by enhancing their surface expression. Our experiments relied on the ibogaine analog noribogaine, which is known to stabilize the inward facing conformation of SERT and the dopamine transporter (SLC6A3) (21, 22). We previously examined other SERT ligands that favor the outward facing conformation or the occluded state and found that they were ineffective in rescuing folding-deficient SERT mutants (20). The fact that pharmacochaperoning is restricted to compounds that stabilize the inward facing conformation of SLC6 transporters indicates that their folding trajectory proceeds through the inward facing conformation (20). It is conceivable that the pharmacochaperoning action of ibogaine or its analogs may be exploited to remedy a disease state resulting from folding-deficient mutations of SLC6A3 (5–7). However, ibogaine is a potent hallucinogen, which limits its clinical application. In this context, it is noteworthy that SERT levels can be increased by treating cells with the chemical chaperone 4-phenylbutyrate (48), which is approved for human use. The chaperoning action of 4-phenylbutyrate on *e.g.* CFTR (ABCC7) is thought to result from changes in the expression of HSPs (40). We recapitulated the regulation of SERT levels by 4-phenylbutyrate and observed an accompanying change in HSP levels, in particular a decline in HSC70 (data not shown). Finally, it is also of interest that single nucleotide polymorphisms in the HSP70-1A gene have been linked to an impaired response to antidepressant treatment (49). Given that SERT is the principle target of antidepressant drugs, it is attractive to speculate that there may be a mechanistic link between variations in the gene encoding HSP70-1A, antidepressant response, and the role of HSP70-1A in supporting folding of SERT.

## Footnotes

3 The abbreviations used are:

ER: endoplasmic reticulum
5-HT: 5-hydroxytryptamine (serotonin)
17-DMAG: 17-dimethylaminoethylamino-17-demethoxygeldanamycin
CHIP: C terminus of HSP70-interacting protein
CFP: cyan fluorescent protein
COPII: coat protein complex II
HOP: HSC70-HSP90-organizing protein
HSC: heat shock cognate protein
HSP: heat shock protein
p23: HSP90 co-chaperone
SERT: serotonin transporter
SLC: solute carrier
VER-155008: 5′-*O*-[(4-cyanophenyl)methyl]-8-[[(3,4-dichlorophenyl)methyl]amino]adenosine
CFTR: cystic fibrosis transmembrane conductance regulator
ABC: ATP-binding cassette.
